# Reliving, Replaying Lived Experiences Through Auditory Verbal Hallucinations: Implications on Theories and Management

**DOI:** 10.3389/fpsyt.2018.00528

**Published:** 2018-10-30

**Authors:** Smriti Vallath, Tanya Luhrmann, Joske Bunders, Lakshmi Ravikant, Vandana Gopikumar

**Affiliations:** ^1^Department of Earth and Life Sciences, VU University Amsterdam, Amsterdam, Netherlands; ^2^The Banyan, Chennai, India; ^3^The Banyan Academy of Leadership in Mental Health, Chennai, India; ^4^Department of Anthropology, Stanford University, Stanford, CA, United States

**Keywords:** voice-hearing, phenomenology, treatment strategies, auditory verbal hallucinations, lived experiences

## Abstract

**Objective:** This study aims to understand the impact of negative life experience (NLE) in auditory hallucinations (AHs) and explain the heterogeneity in phenomenology of auditory verbal hallucinations (AVHs).

**Method:** In depth interviews were conducted with 21 individuals (7 males and 14 females) experiencing AHs and accessing mental health treatment services at a not-for-profit organization. Maximum variation purposive sampling technique was used to select the sample to ensure variegation is accounted for and was collected until saturation of themes data was obtained.

**Results:** Various different forms and functions of hallucinations are obtained with an evident pattern that links voices back to the NLE of the individual. Implications for therapeutic methods focusing on distress arising from said NLE is emphasized.

**Conclusions:** The results obtained from this study implicate NLEs as a contributing factor in the development and maintenance of hallucinations. Sociocultural factors act as a catalyst with psychological factors creating distress and contributing to the voice-hearing experience. Treatment strategies must thus focus on content of voices and past experiences of the individual to promote recovery. A model toward conceptualization of the diversity in phenomenology is put forth.

## Introduction

Voice hearing or auditory verbal hallucinations (AVHs) are a core symptom of psychosis in the Diagnostic and Statistical Manual of Mental Disorders-5th ed. [DSM 5; (1)]. A cross cultural prevalence study found that the 1-year prevalence of AVHs was 74% (2). Although hallucinations can be positive in nature, they are often described as being distressing, intrusive, and impairing one's ability to focus (3), thus making it important to conceptualize and treat the condition more effectively.

Most established non-medical theories of AVHs discuss aspects of cognition, coping, and culture as being factors in promoting the onset of voices. A popular theory notes that dysfunctions in memory and reasoning create difficulties in source monitoring (4) and reality monitoring (5, 6). An externalizing bias has also been established as cognitive underpinnings for hallucinations (7). Yet another theory suggests that an abundance in vivid imagery poses excessive influences on perception, making it difficult to judge internal and external images accurately (8, 9). Consistent with these theories, Waters et al. (10) have observed homogenous results across studies irrespective of methodology, suggesting that self-recognition deficits were pertinent to AVHs in schizophrenia.

These explanations, however, do not account for phenomenological differences in experience. Why do some people, for example, hear their mother while others hear God? One explanation has been provided through sociocultural models that implicate culture in the meaning and characteristics of hallucinations (11–14). Even with this explanation, there remains a gap: why do individuals within the same culture have such diversity in the content of hallucinations?

Recently, research has thus moved from a nosological understanding of symptoms to a more context related, phenomenological and functional understanding, which promotes the notions of psychosocial and sociocultural influences on the manifestation and maintenance of the experience. The formulation of AVHs is thus large, complex, and is influenced by biological determinants (15, 16), personality (17), culture (2), environment (18–20), and lived experiences (21); with these markers evidently noted in the content of hallucinations. In fact, various studies have stipulated that the content and phenomenology of voices can inform therapeutic services (22, 23) and must thus be explored by practitioners.

Despite these recommendations and biopsychosocial understandings of the voice-hearing phenomenon, a causal understanding of hallucinations remains inadequate, especially when considering that the pathogenesis for anecdotes often remain idiopathic. Distress arising from content of voices are also rarely the subject of therapeutic interventions. This study postulates that the most conceivable explanation for variegation in voices may in fact lie in understanding content of voices and lived experiences of the individual; since they influence almost all parts of an individual's life- personality, coping and vulnerabilities. In fact, negative life experiences (NLEs) seem to be prominent among those with AVHs (21, 24, 25). Experiences shape cognitions and schemas that have in turn proven to have an impact on AVHs.

The underpinnings of this notion can be based on the stress-vulnerability models of severe mental illness (26) which suggests that all individuals are vulnerable to developing a mental illness to at least some extent and under stressful circumstances can present as a schizophrenic illness. The authors distinguish between innate and acquired vulnerability; the former refers to vulnerabilities associated with one's neurophysiological system while the latter is one that is influenced by traumas, specific diseases, and other life experiences. This model contextualizes the relationship between trauma and psychosis from an acquired vulnerabilities lens.

Various studies have noted the effects of trauma on psychosis (24, 25, 27, 28) and that stressful life events precede the onset of psychosis. However, only a few studies have focused on NLEs that do not always result in clinical levels of trauma or PTSD, such as death of a relative, abandonment, or even homelessness. In practice NLEs are not the focus of care, since they produce little overt impairment; instead, psychotic experiences take precedence in recovery goals. Similarly, the content of voices which is in fact the source of much distress is often also not catered to. The authors presume that when underlying factors are not managed, the risk of relapse increases. This is conceivably an additional resource and cost to mental health systems, increased disability to the individual and loss of valuable resources to society at large, creating macro and micro burden. Studying the impact of NLEs on content of voices may facilitate formulation of appropriate therapeutic strategies, especially if the event is influencing the psychotic experience and is unresolved.

This study focuses on the relation between NLEs and hallucinations. It aims to explore the impact of said NLEs on the form and content of voices with the premise that they increase stress and therefore vulnerability which can contribute to the experience of voice-hearing. Understanding this relation will influence therapeutic services and social systems of mental health.

## Materials and methods

### Sample description

The current article uses a qualitative approach to explore the reports of voice–hearing. We recruited a mixed sample of individuals who are homeless (IH) and those living with families (LwF). Participants who were diagnosed with schizophrenia or schizoaffective conditions as per the ICD 10 criteria, by a psychiatrist and who claimed they experienced AVHs either at the time of interview, or at least for over a year in the past were included. The experience of AVHs were established prior to the interview by the PI of this study, along with a member of the treating team.

Individuals with an intellectual disability, speech and hearing impairment, hallucinations secondary to brain injury or dementia, alogia, and/or severe formal thought disorders was excluded due to differences in etiology of hallucinations and/or the inability to articulate experiences satisfactorily, as may have been the case.

Pursuing a maximum variation purposive sampling technique, the sample comprised of 21 participants in total including, ten homeless women, six homeless men, four women LwFs, and one man LwF. The homeless men and women had been living in a non-governmental organization (NGO) which specifically caters to the treatment and rehabilitation of individuals with severe mental illnesses. The LwFs group was recruited from out-patient clinics hosted by the same NGO.

The socioeconomic status of the group was low; with the family income being less than INR 10,000 (or 144$) per month. In case of the homeless group the gross income was less than INR 500 (or 7$) per month. Most individuals in this group were working as daily laborers completing different chores either within or outside the organization the study was conducted in.

At the time of the interview, 16 participants were symptomatic of their respective diagnosis (showed florid symptoms, especially hallucinations), four participants were in partial remission (showed some symptoms at the time of the interview, but not severe enough to affect functioning) and 1 participant was in complete remission. Participants had varying levels of insight ranging from Grade 1 (no insight) to Grade 5 (intellectual insight) that was established by the PI who is a psychologist and members of the treating team using qualitative information to ensure nuances related to insight were not lost that can often be the case with quantitative tools. The level of functioning as per the global assessment of functioning (GAF) scale (29, 30) ranged from 41 to 63 (Disturbances in three areas of functioning to mild and persistent symptoms and mild difficulty in social, occupational or school functioning). This was assessed by the principal investigator during the phase two of this study (as described below), to gain a more holistic understanding of the participant and their AVHs and NLEs.

### Procedure

#### Phase 1

NLEs were operationalized experiences that create negative emotions such as sadness, loss, distress, anger, resentment, or mental confusion. In depth interviews were conducted with each participant by the principal investigator using a semi structured interview schedule (see Supplementary Material). The interview schedule used by Luhrmann et al. (13) to explore voices was adapted to suit this study. The focus remained on the following dimensions of AVHs: rate/frequency/senses; form and relationship; control; realness; perceived cause; subjective distress; positive outcomes; other associated symptoms and on understanding lived-experiences (interview schedule: appendix). However, not all participants were able to produce reliable information on lived experiences. This was therefore managed primarily in phase two of this study. Each interview was 30–60 min long, conducted in the regional language of the participant and were manually transcribed by a professional.

#### Phase 2

Case records of each participant was reviewed to explore life histories and other details of hallucinations. Respective case workers were engaged in this process and an informal discussion of the case was conducted with them to gain a more holistic picture of the participant. This revealed some more anecdotal evidence that supported the findings of this study.

On completion of the first ten interviews, analysis commenced, while data collection continued until saturation was obtained (*n* = 21). Saturation was achieved when themes were recurring from participants and no new information was evident, rendering further data collection redundant leading to “informational redundancy” [e.g., (31)]. Analysis was primarily completed by the principal investigator, and was reviewed by some of the other authors of this study at the end of data collection.

### Data analysis

Each transcript was uploaded on a qualitative software, “Dedoose” V 7.0.16, which was used to code. Codes were developed inductively with each transcript, using a line by line approach, until saturation was obtained. The framework for analysis was inductive toward description of life experiences and voices and functions of the voices. Themes were then chosen by grouping codes together that indicated a match to a superordinate theme, which were in-turn related to life histories obtained from case managers and case records. A psychological and sociological approach was taken to interpreting themes, by linking life experiences to existing principles and theories in psychology and sociology. The last author of this study then reviewed the themes and its interrelation with life events obtained from case workers and patient records, to corroborate findings.

## Results

**Figure 1:**
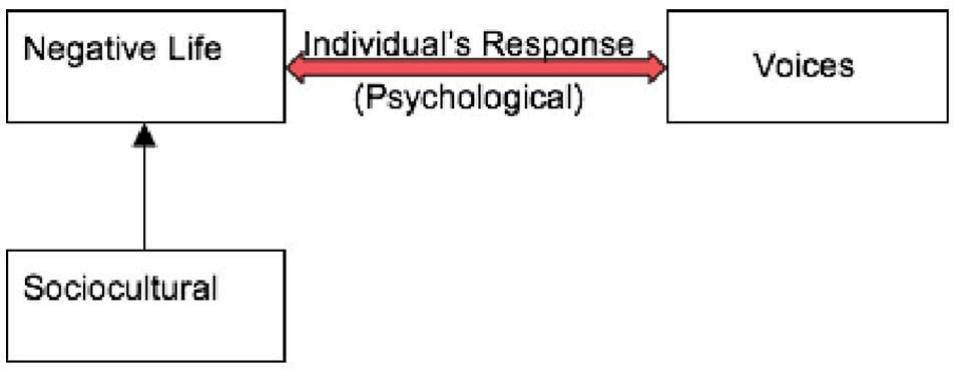
Showing the interrelation between the sociocultural context, individual's response (psychological) and NLE in voice-hearing.

Findings revealed that voices are similar to one's NLEs which stem from the individual's sociocultural and psychosocial contexts, such as dysfunctional family, marriage, social control, social schemas, loss, abuse, or failure. The individual's subjective appraisal of the event or NLE often determined the content of voices. For example, an individual may appraise an event such that it results in feelings of loss or abandonment which in turn can play-out in the content of voices. Voices therefore parallel individual's response (distress) to the NLE (see Figure 1). This will be further elucidated in the sections below under superordinate themes.

The next sections will elucidate on the findings obtained beginning with a description of the form and structure of voices in section A, then the function or purpose of voices will be described in detail with quotes from participants and ending with a case study that further establishes the function of voices.

### Section A

This section focuses on results obtained that are related to the form of the hallucination; various sub categories were obtained.

The **FORM** of hallucination is operationalized here as the shape or structure the voices take, as perceived by the experiencer. The form of the hallucination is significant and instituted in one's life experience and culture. It could be demarcated into two broad kinds: the supportive and the derogatory which could further be divided into two categories that include religious and sexual connotations respectively, each with a different emotional value to the experiencer and each with functional properties, as will be elucidated in the subsequent sections:

1. **Supportive and derogatory Voices:** Supportive voices are those that were perceived by the experiencer as being kind, loving, caring and were associated with positive attributions “… They [the voices] say that I will get a good job…” Said Priya, similarly, “*…an elderly man with beard talks… he says nice things… he is ‘Allah’…”* while derogatory voices were condescending, rude, or those that made the experiencer uncomfortable, often causing distress “*…They say [the voices] that my son is dead…”* said Devi. Another participant expressed “*… only ‘Raja’ says dirty [sexual] things to me…others don't…”*

Three participants experienced derogatory voices that often spoke about their bodies, sex, masturbation or their shortcomings; while six individuals heard supportive voices. The remaining participants (10) heard both supportive and derogatory voices. For two participants the voices were unclear and content was therefore not demarcated into either category. It was especially interesting to note that while there were individuals who heard only supportive voices, except for two participants, all those who heard derogatory voices also heard supportive ones concomitantly; Supportive voices offer hope and support in a state where the derogatory voices cause distress as can be seen in quote below:

Leela said “…yes she [the voice] talks about my body…I can't even say what she talks [exactly], it's such foul language… [later in the interview] God talks to me decently. I ask him something and he answers [politely].”

Further, respondents claimed that derogatory voices were heard in third person, and were identified as those belonging to a woman or the larger community while supportive voices on the other hand, were from God, friends or parents.

- Sexual content:

Content of voices had sexual connotations for eight clients. Such voices are considered derogatory and cause anxiety and distress, such as in the case of Teju who hears voices of her neighbors and relatives “*…they talk all the time. When I have sex with my husband they talk about it, they talk about my body when I am taking bath. I have no peace of mind. I have stopped having sex because it's too embarrassing…”*

- Religious Content:

Voices with religious connotations were observed in seven participants. Religious voices often gave the experiencer a sense of hope and calm such as in the case of participant Prerna who reported hearing Allah:

“*…he asks me not to worry. [He] says he will take care of me…”*

**2. First and Third Person Voices**

Interestingly, 14 of the 21 participants heard voices in only first person. The remaining seven participants heard voices in both first and third person. No single participant heard voices only in third person.

Participant Mustafa, 48-year-old, homeless male, diagnosed with schizophrenia, hears voices in both first and third person-“*…yes they call me for interviews…they also discuss it [job openings] among themselves…”*

Participant Shreya, 50-year-old female, diagnosed with schizophrenia hears voices only in first person-“*my son gives me directions when I am traveling alone so I don't get lost…”*

**3. Gender of Voice**

It was also found that only six participants heard male voices alone.

“*Yes they will shout at me and say work, we're working…”* said Hari a 30-year-old male diagnosed with schizophrenia, LwF.

All remaining participants heard both male and female voices.

“*My brother talks to me and tells me he will take me to my parents…My grandmother consoles me*…” reported Tina, 45-year-old homeless female, diagnosed with schizophrenia.

It was a curious finding that no participant heard female voices alone. It was also noted that among those who heard both male and female voices, the content of what the former spoke was that of power, kindness, and supportive; whereas the latter (female) voices were those that were demeaning, insulting and rude; irrespective of the experiencer's gender.

**4. Recognition and Age of the Voice**

Eighteen of the twenty-one participants were able to recognize the voice as being somebody they knew like a God, a powerful and popular leader, most often a relative or friend;

“*…Yes [I hear God] …he talks to me about the war in Israel…”* said Manoj, 35-year-old homeless male, diagnosed with schizophrenia.

For nine participants the voices were those of someone older than them, irrespective of whether they knew the person or not. Three participants reported the voice belonged to someone younger than them. Two participants were unable to attribute age. The remaining participants heard multiple voices, some of whom were of people younger than them while others were older than them.

**5. Engagement and Control with the Voice**

18 participants reported engaging with the voice, i.e., they initiated and maintained conversation while three avoided engagement. Irrespective of engagement however most participants (18) were not able to control the experience of voice-hearing.

“*…I have [tried] but they ask me to go die…”* Said Shika 55-year-old homeless female, diagnosed with schizophrenia

“*…no ‘[I don't have control] I will have to hear them till I die…”* Said Prerna, 45-year-old, homeless female, diagnosed with schizophrenia.

Two participants reported to be able to control them:

“*I can speak to them whenever [I want to] … like when I am lonely…”* reported Anna, 60-year-old, homeless female, diagnosed with schizophrenia.

“*…if I have time I choose whom I should speak to and when…”* said Manoj, 35-year-old, homeless male, diagnosed with schizophrenia

One participant reported she could control some of the voices sometimes but not always and not all voices.

### Section B

#### Function

The findings revealed that voices served subjective purposes to the experiencer. This section will focus on the function of hallucinations, that were developed as themes from the data obtained and interpreted from psychological and sociocultural perspectives. The content of hallucinations was key in determining functional properties of the experience, irrespective of the form i.e., derogatory voices, for example, could have a positive impact on the individual. The function of voices was established for all participants except in five for whom it remains unidentified. This was likely due to inadequate information in case histories since they were individuals who have experienced homelessness and/or no reliable informants are available.

Three broad themes were obtained:

- Voices were a **Reflection** of the person's NLE- i.e.; they were directly related to a participants' life experience.
- Voices helped in **Coping** with NLEs

Although the methodology used in this study does not permit to establish causal attributes; triangulation of data has provided evidence for the argument that AVHs may in fact provide support or/and reduce levels of anxiety by enabling different processes such as venting, replaying different scenarios to problem solve, and receive a “push” depending on how the experiencer has appraised an event or need in their lives.

Thus, for six participants, hallucinations seemed to be a reflection of their NLE; for five, the experience could be seen as a coping mechanism. For another six participants the voices were both reflections and coping mechanisms. The remaining four participants were unable to describe the content of voices sufficiently to reach conclusions of functions and have therefore not been categorized into either purpose. The sections below illustrate the different themes, obtained.

1. **REFLECTION**: A direct thematic relation was established between the experiencer's NLE and the AVHs. Some of these were analyzed as emerging from specific psychological processes and served different purposes, as described below.
  - Cognitive dissonance is the mental stress experienced by a person when s/he holds two or more contradictory ideas, thoughts, values or beliefs (32).

This was apparent in participants as in the case of Tanya who reported that she was sexually assaulted in the presence of her father who later, also went on to sexually abuse her. She however believes that for a child the father is important and can do no wrong. She reported that despite all his doings he was a “good father.” Tanya hears the voice of her dead father, verbally assaulting her using foul language; as a reaction, Tanya also responds using foul language, often calling him a “useless father.”

“…He calls me mad and that I had a dirty fu^***^ng husband and I was born to his first wife. And that I didn't know about any crap…How can he send a young girl inside and leave her alone with a man; he doesn't even know that much! [later…] no, when a father talks to a child how can she not like [it]; it's okay. He's my father he can shout at me…He was [a good father] …He bought me anything I asked for…”

Similar instances were noted in the case of Mathew who reported he heard the voices of more than 10 people, all of whom were fighting against each other. They belonged to two different religious groups and were trying to get him on their side, respectively. It was later discovered that Mathew's mother had converted to Christianity from Hinduism while he was a young boy. He expressed that he had felt very conflicted because he was always told that he should pray to only Hindu Gods but then one day he suddenly began attending a Christian church.

“*…they fight among themselves because they're two different groups and they both want me on their side. But you tell me is this possible? They punish me for praying to Lord Shiva instead of Lord Murugan or sometimes they bother me if I pray to Jesus.”*

- Inability to maintain social expectations

Another instance when voices reflected life experience was when the experiencer could not live up to social expectations and lacked social support. Participant Hari for example, heard voices of his coworkers shouting at him, urging him to work, while sometimes providing him with guidance when faced with a task he could not complete on his own:

“*Someone will shout at me…like, while I am sitting here [working] someone will talk to me…[they say] do work properly… they shout… [it's okay] they guide me [making me work well]…”*

In another example, Vishal a 30-year-old male hears the voice of his brother-in-law being supportive of his limitations and offering words of encouragement.

“*My brother-in-law is also a very nice person…he tells me not to worry and that everything will be alright… he said I should live my life and if necessary he will get me a job…I feel nice, like good will come.”*

Similarly, Rama, a 30-year-old, LwF woman, diagnosed with schizophrenia, reported that she heard voices of a man, a child and her father. The voice of the child often called out to her for food. It was later learnt that Rama felt guilty about having neglected the child during her episodes. She reported that the voices caused her distress because the sound of the child crying was unbearable.

“*… I used to hear a child's voice asking me to do things for it and it used to call me mother.”*

2. **COPING**: Coping in this study is operationalized as methods used to manage those events and associated emotions or feelings that cause distress. Voices can be seen as being helpful in managing demands the individual has to meet, as a response to some events. Below are some thematic clusters of coping mechanisms used, obtained from the data.

- **Wishful thinking** is a process of belief formation and decision making according to what one might find pleasing, instead of applying evidence, rationality, or even the paradigms of reality (33).

In the case of Priya, for whom the voices were a source of support during difficult times, when she faced physical and emotional abuse.

“*…I was young, when my father and uncles found out [I applied feces on myself to look prettier and become stronger]. They began beating me. They didn't even care that I was a girl and naked, they were beating me. I was a small girl only, I know. But still I was naked and everyone was looking at me; everybody from around came. It was so bad. Later my friends [voices] asked me “why didn't you call us, what if something had gone wrong; if they had killed you who would be answerable! You should have called us….”* She reported feeling supported and like “*someone is there.”*

Similarly, Manoj reported that he hears the voice of various high-end political leaders such as “Obama” and “Putin” talking to him. They ask him for advice regarding their political careers and so on. It was later learned that he was an extremely “carefree,” lucky-go natured boy whose parents were always pressuring him to achieve more. Manoj's parents always accused him of being “good for nothing” and “careless” so much that they refused to arrange a marriage for him calling him “irresponsible.”

“*…the voices talk about my family, problems in the village, problems in Tamil Nadu…Obama speaks to me from a box…I know 56 presidents and speak to a few of them, Hitler, Putin… they speak about their country's problems and about the South-Asian Conference…”*

Shreya, who reported that she was sexually abused as a child and was raped multiple times hears the voice of a policeman who acts as a crusader for the abused and battered women, whispering words of courage and encouragement to her. Her need to punish her offenders and save herself, while being helpless is seen as being projected upon a fantasy strong, powerful man who can help her fulfill what she cannot achieve on her own.

“*Das police will help. He has helped many women like me. He even told my husband he will give money to keep me but it didn't work. He says he will catch all the bad men and protect me. He has always been there for me and protected me well.”*

**Source of Comfort and Hope**: The voices sometimes provided dialogues that were comforting and eased anxiety for the experiencer. Anxiety often stemmed from ideas related to the NLEs of the individual; like in the case of Prerna who heard the voice of God offer support while other voices ask her leave the center she stays in. Prerna was asked to leave her home by her family [reasons unknown].

“*…an elderly man with beard talks… he tells good things… I don't know who he is, but I am able to see him, he is Allah. Like father…”*

Anna, also reports that she hears the voices of her relatives living in The Philippines, who talk to her and help her feel “connected.” Her instinctual need to fight loneliness can be seen as displaced on her voice hearing experience, thus reducing anxiety.

“*…they ask me whether I ate or if I've rested well… they say they miss me and look forward to meeting me soon…It's nice that there is somebody to ask me these things after all these years.”*

**Figure 2:**
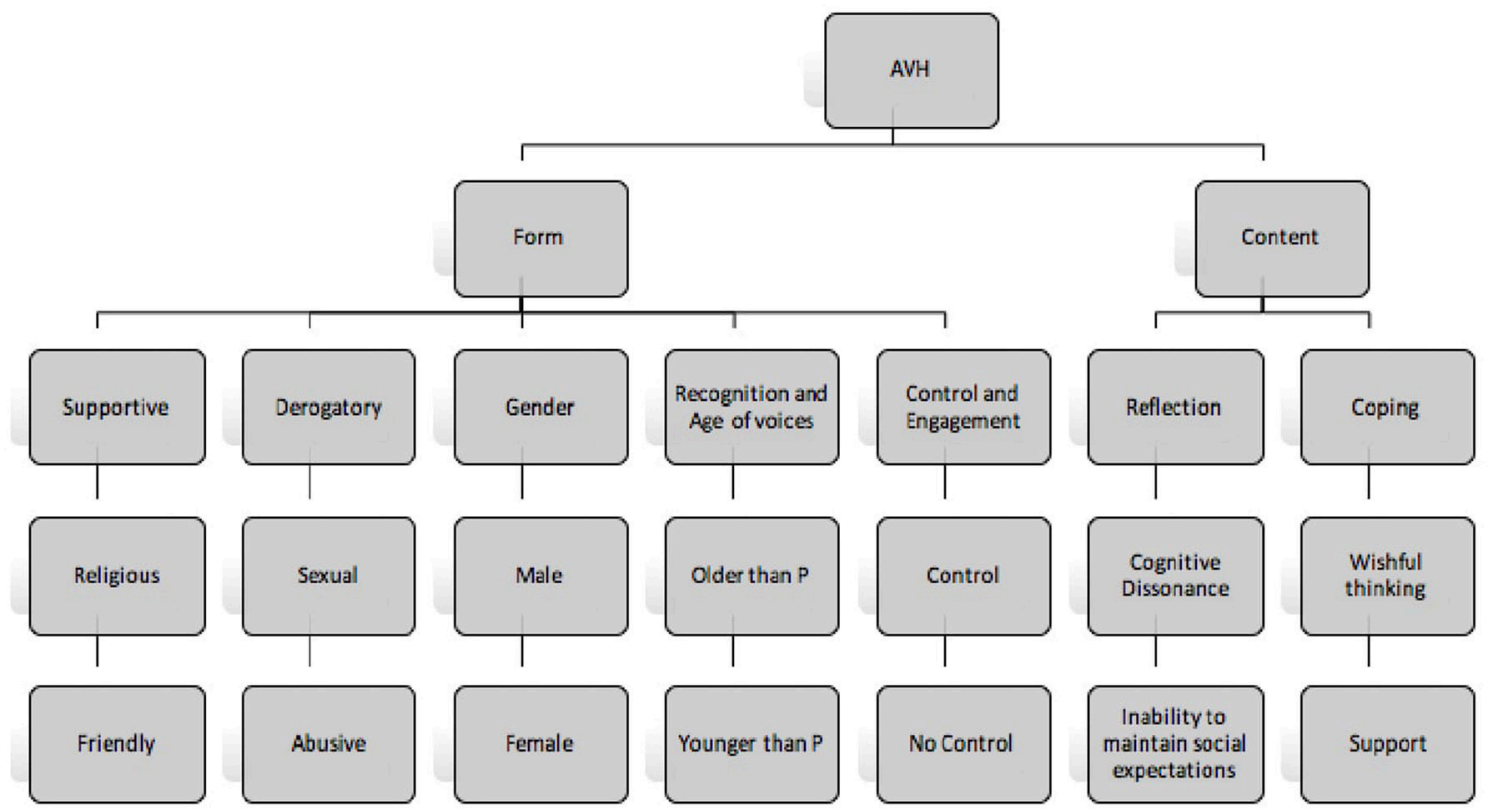
Diagrammatic representation of the results obtained.

Figure 2 presents a summary of the aforementioned themes. The case study below is a prime example of the function of voices stemming from NLEwhich may be contributing or perpetuating factors. It sets the context for further discussion and provides conclusive evidence that voices serve a purpose and identifying that purpose may be key in recovery goals.

Mustafa is a 45-year-old male diagnosed with schizophrenia. He hears the voices of dignitaries from the national institute of mental health and neurosciences (NIMHANS), Bangalore, India call him for interviews to pursue his MD in Psychiatry. He believes this is truly possible and often asks those around him to facilitate the process of rejoining school.

Mustafa lives in a shelter home facility for men with psychosocial disabilities, after a period of homelessness. He speaks impeccable English but lacks coherent thought. To any person listening to Mustafa, the description of his voices seem unrelated to his current life. Mustafa's distress and feelings of helplessness seem secondary to psychotic thinking and nothing more.

However, when his history is considered, the voices make more sense and can be situated within personal context… Mustafa was given up for adoption as a toddler, by his biological mother, to her sister (Mustafa's aunt), when they faced socioeconomic problems. Mustafa was then taken away to Dubai where he led a happy, pampered life, ignorant of his history of adoption and that status of his biological family back in a rural neighborhood in India.

It was in his late childhood-early teenage-years that Mustafa learned about his adoptive status. Details are unknown around this area, but the family and other mental health professionals who work with Mustafa recollect that it was around this time, at the age of thirteen that he began having problems. Mustafa began using alcohol- a behavior he learned from his adoptive father who consumed alcohol on a regular basis. He also began having behavior problems that made it difficult for them to manage him. He would often have temper outbursts, throw tantrums and be physically aggressive with his playmates and others. Mustafa's father was authoritarian in nature and very strict. He would also react aggressively to Mustafa, only worsening the problem.

With this situation at hand, his adoptive parents decided to send him back to India to live with his biological family. It's here that he lived until the age of seventeen, when he returned to Dubai. Not much is known about his years in India but the family recollects him having “changed.” He no longer used alcohol or had aforementioned behavioral problems. As always he remained an excellent student and at the age of 20 was sent to a medical school in Bangalore, India. Mustafa recollects his passion for computer sciences but a medical degree was considered more valuable by his father and was thus insisted upon. Mustafa was told that without a medical degree a man could not lead a successful life. He therefore agreed, though reluctantly and moved to India for his education.

At the medical school, Mustafa got friendly with other international students and was often using substances such as alcohol, marijuana, and other drugs. He also indulged in casual sex and other high risk behaviors. He however, maintained his grades and made it into his fifth year of school.

He was 25-years-old when he had his first psychotic break. He remembers preparing for his medical finals when he was caught red-handed for using drugs. He was facing an inquiry regarding his conduct at the same time. He recollects this period being particularly stressful. He was also called for an interview to NIMHANS for his MD in psychiatry. He completed his exams and was attending the interview in which he claims to have been selected to pursue his MD. Hearing the good news, he left for Dubai the same evening, without knowledge of results from the drug inquiry he was facing.

On reaching home, he was excited to tell his father the good news. However, the home was filled with grave silence. As the first words came out, his father slapped him hard on his cheeks for having been expelled from medical school on accounts of drug use. This was the first Mustafa had heard of the news and was shocked. It was at the same time that his father said “you proved today, you're not my son!” Since then Mustafa isolated himself from his adoptive parents, stayed in his room, alone and would resort to violence in case they approached him.

They therefore sent him back to India, again, to his biological family. Here, he never felt at home and would often leave. During his episodes of homelessness, he would use substances and then return. It was during one such episode that his mother attempted to stop him, when he beat her. His biological brother not able to handle himself, beat him back and asked him to leave home. Since then began Mustafa's descent into homelessness. At the age of 35 he was found and his family sought treatment at NIMHANS, in vain since his compliance with drugs was poor. He showed poor prognosis and continued to be difficult to manage. At the age of 42 he was taken to the ‘beggar’s home' where he met his psychiatrist by chance. He was then referred to an NGO in Delhi, near his hometown but continued to be difficult to manage. He was moved to the NGO in this study in 2015 and has continued to stay.

The above case study narrates the complex nature of hallucinations that not only reflects one's NLEs but also one's deepest, and perhaps unfulfilled desires. Mustafa's father's ideas of medical school being important for a man seems to have created an imprint. The voices Mustafa experiences do not seem out-of-context when one explores this case study. It is evident that Mustafa carries feelings of loss, failure and inadequacy resulting into distress. In coping with the distress, he hears the voices of doctors from NIMHANS call him for interviews and to secure his future:

“*They call me and ask me to come attend the interview for my MD…sometimes they also ask me to sit on the board and select future candidates…”*

## Discussion

The current study examined the nature of hallucinations, its phenomenology, purpose and possible contributing factors. As is common knowledge, a dominant view in biomedical approach to mental health, which suggests that conditions such as AVHs are meaningless and simply a manifestation of an underlying biological imbalance (34, 35), this research finds that hallucinations are often meaningful and relate to one's psychological and sociocultural context, particularly the experiencer's NLEs and related distress. NLEs were noted to have a substantial impact on voice-hearing experiences. This is consistent with previous findings that suggest voices are exacerbated because of stressful life events (36) and that life events were linked to the prognosis of the illness (37).

Voices were found, to not only be meaningful but also have functional properties, especially in coping with the NLEs; which seem to not only have contributed to the experience but also perpetuates it. Even, if they were just reflections of the negative event, triangulation of data suggested that they helped in venting unresolved issues such as in the case of participant Tanya, or offer comfort such as in the case study of participant Mustafa. Further, the form of the voices also suggested a “balanced” mechanism: supportive voices occurred concomitantly with derogatory voices, thus reducing the level of distress from derogatory voices.

It seems, that during a state of crisis, such as an unmet need, an individual adopts certain mechanisms to restore emotional balance like fantasizing, in the case of participant Priya, or as elucidated in the case study of Mustafa. Such “immature defenses” though may seem pathological, serve as the brain's homeostatic efforts to cope with these changes (38). In fact, Folkman et al. (39) emphasize that coping strategies must be evaluated in context, since their appraisal depends on how they meet situational demands. It seems that in certain cases, wishful thinking, though hallucinatory in nature, tends to offer a sense of support, thereby meeting a situational demand. Moreover, the finding that the function of wishful thinking is to cope with NLEs, is consistent with findings in another study which found wishful thinking to be associated with avoidance symptoms of PTSD and chronic PTSD (40). Similarly, another study has noted that the phenomenology of voices in psychosis and PTSD are in fact similar (41, 42), which further supports the hypothesis made in this study that NLEs have a significant role to play in the construction of voices.

This study postulated that NLEs are related to the content of voices. On finding convincing results, it became important to understand more specific factors that interplayed between NLEs and voices. Life events were thus analyzed in view of existing sociological, psychological and biological theories and are presented below:

### Social factors interplay in voice-hearing experience

Social factors are operationalized as those that have been maintained by communities and groups in societies to maintain the notion of social order, which is usually instituted through social norms. Social norms are attitudes in society, of approval and disapproval for what can be accepted and not accepted (43) so that the group at large is benefited. Deviance from norms and its consequences tend to affect individuals, can be intense, and can cause unpleasant feelings (43). This understandably influences social cognition- which refers to how people think of themselves and others in the social world (44), particularly the cognitive and emotional functions necessary to predict another's mental state or behavior (45) which in turn has been linked to different positive and negative symptoms of psychosis, in various studies (44).

With this information in hand, it is only conceivable why participants like Hari heard his co-workers shout at him for not working or why Rama heard the voice of children or why Mathew had difficulty coping with the change in religious practices. Social norms and customs insist on particular behaviors and attitudes in life; when said behaviors are not practiced, associated networks often reprimand or reject.

Influences of social control were noted in most participants of this study, and deviance from it, even when not deliberate, resulted in rejection and neglect very early on in life. Conceivably this has a psychological impact. Most individuals are motivated and strive to conform (46) to either fit in (normative influence) or because they believe the group is better informed than they are [informational influence; (46, 47)]. Individuals feel the need to belong with different social groups—this need for affiliation (48) can have a positive impact on well-being (49). However, when individuals deviate from norms of social control, the rejection, abandonment, and associated disaffiliation, can understandably led to distress, as seen in the case study of Mustafa; which can in turn make an individual more vulnerable (26) to psychotic experiences such as hearing voices.

Deviance from social norms was not the only time social factors seemed to interplay with the voice hearing experience. The form of the hallucinations was demarcated into two clear types—supportive and derogatory. It was interesting to note, that often religious voices were considered to be supportive while sexual voices were considered derogatory. A social link to Indian social norms has been identified. It remains a taboo to talk about sex in public or one's sexual activities with others; if ever spoken, it's hushed and always in confidence. Often even legitimized sexual activity requires permission and must have procreative value. In fact, various insults used during confrontation or conflict, in the local language insinuates sexual activity. It is thus not surprising to note that experiencers report sexual voices as derogatory and experience distress.

### Psychological factors also interplay in voice-hearing experience

Psychological factors here, are operationalized as feelings, thoughts or behaviors that can affect an individual's optimal functioning. As mentioned above, social control and the need for affiliation was seen among all participants; deviance from social norms leading to stress was also evident. It was found that failure, stress, loss, trauma, and unmet needs were particular psychological factors evident in life histories leading up to the onset of hallucinations. The cognitions and schemas resulting from these factors included those of guilt, loneliness, feeling unloved, creating an inability to cope.

In situations, where there was a perceived lack in support or other mechanisms to help manage the event, it seems possible that individuals develop hallucinations to serve that purpose, however this has not been conclusively established due to the nature of this study. As seen in the results section, the case of Tanya who could vent negative feelings she had for her sexually abusive father, or in the case of Shreya whose feelings of victimization lead her to hear the voice of a crusader, out to help women like her; or in the case of Tina who felt so abandoned that she heard the voices of her brother and grandmother consoling her and promising her to bring her back home. Such anecdotal evidence is in abundance among participants of this study.

In dealing with difficult and complex emotions, especially where resolution is difficult to attain, some individuals retreat into using regressive coping strategies and experience hallucinations as a medium to play out cognitive dissonance or other conflicts and negative emotions. This notion has been sustained in psychodynamic theories which suggest that hallucinations are a breakthrough of the preconscious or unconscious material into consciousness in response to situation or needs such as wish fulfillment (50). Similarly, Corstens et al. (21) for example found that 94% of their sample experienced underlying emotional conflicts that the authors formulate as being played out through voices. Further, various theories have discussed deficits in monitoring internal and external events among individuals experiencing hallucinations (51, 52). This is further made central to this paper when theories of intrusive thought are explored. Intrusive thoughts are often negative, ego dystonic (53) and triggered by external events (54). A stark similarity is noted between hallucinations and intrusions, with both being egodystonic; indicating a strong relationship (55–57). It is thus conceivable to formulate that negative life events that are unresolved continue to foster negative emotions which are intrusive and contributing to voice-hearing. Further, this also explains the influence of stressors and triggers on relapse or exacerbation of voice-hearing- since intrusions are triggered by external events.

These explanations substantiate findings of this study where voice-hearing experiences of most participants was precipitated by stressful life events. Further, an evident finding was the prevalence of perceived unmet needs from childhood or one's past. Most individuals reported either in the interviews or in their case notes that there were various needs from the past had not been achieved which continued to cause distress in the here and now. It appears that most individuals used regressive coping strategies such as avoidance and/or wishful thinking; these have often been linked to lower levels of satisfaction (58) or resolution.

### Role of neurochemical factors

Though beyond the purview of this paper, a brief review of the neurochemical factors involved in stress reveals linkages to dopamine pathways in the mesolimbic system (59, 60) that has also been implicated in schizophrenia and other psychotic disorders (61, 62). This implicates the role of biological factors in the origin and maintenance of AVHs.

The section above has distinctly elucidated the role of social, psychological and biological factors in voices. The section below discusses the way forward from the current discussion and drawing implications for future research and practice.

### The way forward—diverse possibilities and scope for future research

There has been ample evidence suggesting an interplay of social, psychological and biological changes leading to hallucinatory experiences (63, 64). While the neurochemical changes are moderated with antipsychotic medication (APM), which is the primary and sometimes the only mode of treatment individuals receive; this study finds that psychosocial factors stemming from NLEs also requires attention. Various studies have noted a positive history of trauma in individuals who have a diagnosis of a psychotic disorder (65, 66), such as hallucinations.

In fact, recent studies have been attempting to delineate a complex set of inter-relationships between trauma, psychosis and post-traumatic stress disorder [PTSD; (67)]. This study holds that trauma is a subjective perception and must be measured by the intensity and severity that an event poses to that individual. In a study by Gilmoore et al. (Unpublished) it was found that within the Indian context individuals reported more events as traumatic than those noted by the DSM 5 (1) or ICD 10 (68). Within this context, this study finds that not only traumatic events but even NLEs are closely related to voice-hearing. The aforementioned stress-vulnerability factors (26) supports this notion. Distress is therefore a revolving door between hallucinations and recovery and is seen at the origin of the experience, beginning with a NLE, and is also one that maintains it. A similar idea was put forth by Thomas et al. (69) who claimed that stress is an antecedent to voice hearing and the voice-related distress may result in a “maintenance cycle” of the experience.

Psychotherapy must thus focus on treatment of trauma/NLEs while treating psychosis. The design of this study does not allow for causal inferences to be drawn. But it is clear that there is an association between NLE, distress and hallucinations as discussed above. Many recent studies also advocate for trauma-based interventions (42, 69–71). In fact, some authors have suggested that dominant interventions like CBT do not particularly help resolve malevolent voices (70, 72) that are often associated with trauma.

**Figure 3:**
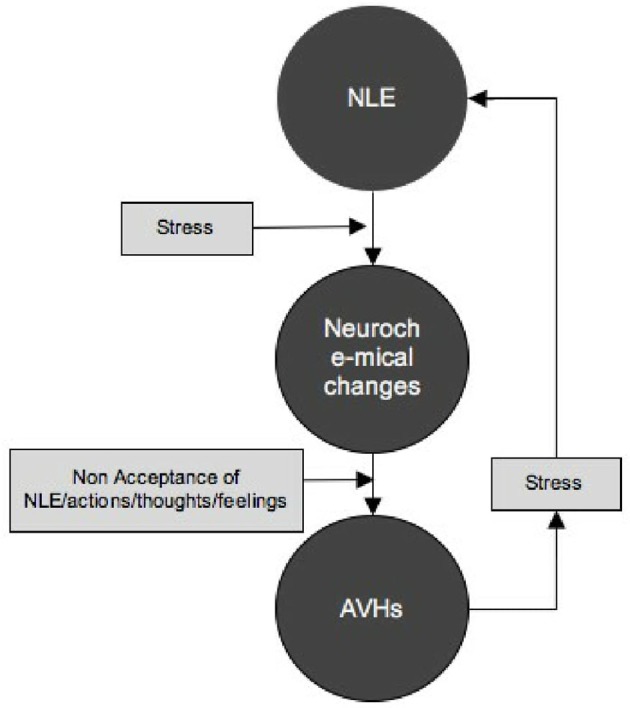
Showing the link between NLE and AVHs as developed through the findings of this study.

With that, a cyclic approach, grounded in a biopsychosocial model is proposed, that also accounts for individual differences (Ref. Figure 3). Firstly, life experiences (positive or negative) impact the individual; however, NLEs have more impact on the individual's psychological state. This is called the “negativity bias” and has received substantial evidence through various studies (73–75) even at the neuronal/brain level (76–78). These NLEs are managed through coping mechanisms, which may have dysfunctional attributes in theory, but in practice have promoted survival for the individual at a given point in time. Secondly, the stress induced from the NLEs causes neurochemical changes in the brain, that thrust hallucinatory experiences in full force, and thus an onset. Thirdly, the initially experienced NLE acts then as a catalyst to this neurochemically charged brain which induces memories that are attributed as externally arising due to motivational factors as mentioned by Morrison et al. (55). Fourthly, the stress/distress experienced as a direct result of the AVHs or from the implications of such an experiencing in a society such as stigma or decreased functionality causes stress which acts as a NLE in itself, which further releases neurochemicals, maintaining the AVHs. This was also noted in the results obtained in this study, where most participants, irrespective of the type/kind of voice (supportive/derogatory) experienced distress and wished it would stop.

Considering the variety of NLEs, differences in social environment and psychological coping mechanisms it is now conceivable that subjective differences in content of hallucinations is in fact unremarkable. Various theories have suggested the implication of coping at the ego level which is aimed at protection of personal meaning or ego identity from existential crisis (79). Unlike previous theories of AVHs, this explanation goes a step further and explains why only certain experiences (negative) are reflected in AVHs and not others (positive).

## Implications and conclusions

The results obtained form the basis for a re-conceptualization of AVHs in clinical practice. The content of AVHs is evidently important and significant, often reflecting the starting point for therapy—events, emotions, actions, or thoughts. Non-resolution of NLEs and its associated factors, contribute to stress/distress, maintaining the voice-hearing experience. Considering this it is plausible to postulate that psychotherapeutic strategies focusing on helping experiencers come to terms with their NLEs, events, emotions, actions or thoughts, may impact the experience of AVHs as well. Psychosocial interventions that focus on content of AVHs and formulating connections to one's lived experiences may prove to be an important factor in treatment strategies, adjunct with neuroleptics. Therefore, therapies such as cognitive analytical therapy (80) or Narrative Exposure Therapy (81), which help manage NLEs in addition to trauma may be more helpful than those that focus on the manifestation without managing underlying factors.

Further, post management of NLEs, the social devaluation that the individual has experienced maintains distress which may in turn cause an NLE again. Toward this, helping individuals with attaining social roles and pursuing capabilities may help break the cycle of distress, NLE, and voice-hearing. The individual is therefore not only taught to manage emotions associated with NLEs but also to change reality to better suit their identities as they see fit.

The theoretical model put forth above, could be the way forward for future research. This can be tested using a randomized controlled design to test validity of the model, which this study could not complete due to logistical limitations. However, instituting the understanding of such results in practice remains key in improving referral pathways to strengthen through early identification and prognosis through interventions for NLEs.

## Strengths and limitations

The paper has elucidated in detail through the multiple sections above the importance of the content of voices, its relation to NLE and implications for therapy. The paper provides a way forward for researchers to unify the biopsychosocial approach in explaining individual differences in content of voices and the need for care packages to adopt this approach in conceptualizing cases and developing care plans.

1. Readers must be aware of possible retrospective bias and memory distortions in some cases; considering the nature of the study.
2. We think a longitudinal study focusing on the types of NLEs, factors that impede coping and therapies that can be used once the incident has occurred will have preventative and treatment implications.

## Ethics statement

This is a study that is aiming at understanding some of the voices you hear. Knowing more about your voices can go toward helping us understand the problem and thereby build care packages for you and others who experience similar problems. All your responses during this research will remain confidential and will not be shared with your treating team, without your permission, unless there is cause for concern. Most questions are simple and should not bother you; however, in the event that you feel they are bothering you, please let us know and we can either stop the interview and continue later, or terminate it. You will not lose any of your existing privileges because of any event that happens during the course of this interview. Please note that for the purpose of accurate documentation and analysis of your responses, they will be audio recorded and transcribed by a third party, who will ensure nondisclosure. This research has been vetted by The Banyan's ethics committee to ensure that your rights and privileges are preserved. We have taken great care to ensure that no harm befall you during or because of your participation in this research.

## Author contributions

SV is the principle investigator - she was involved in conception of the idea, formulating it and designing the study; with mentorship and supervision from TL, JB, and LR. TL provided the interview schedule and many helpful comments on the various drafts. JB helped engage with results and carry the research forward ensuring best practices, as well as provided some helpful comments on the draft. LR was involved in structuring the articles, providing many helpful comments. VG collected data with the PI on occasion, helped SV engage with results and offered many helpful comments through the various drafts of the article. VG was also involved in supervising SV during data collection and analysis.

### Conflict of interest statement

The authors declare that the research was conducted in the absence of any commercial or financial relationships that could be construed as a potential conflict of interest.
