# Supplementary material for: Reliving, Replaying Lived Experiences Through Auditory Verbal Hallucinations: Implications on Theories and Management
Source: Front Psychiatry. 2018 Oct 30;9:528. doi: 10.3389/fpsyt.2018.00528 (PMC6218596; doi:10.3389/fpsyt.2018.00528)
Supplement: Supplementary file 1 [file Table_1.DOCX]

**Supplementary Material**

INTERVIEW PROTOCOL- Luhrmann et.al., 2015; adapted.

**Rate/Frequency/Senses:**

- How many voices do you hear; how often do you hear them

(many times an hour/ many times a day/once a day/once a week/less often)?

- Do you ever hear other things? Bangs, scratches, bells? How often?

(many times an hour/ many times a day/once a day/once a week/less often)?

- Do you ever see things that other people can’t? how often?

(many times an hour/ many times a day/once a day/once a week/less often)?

- Do you ever smell things that other people can’t? how often?

(many times an hour/ many times a day/once a day/once a week/less often)?

- Do you ever taste things that other people can’t? how often?

(many times an hour/ many times a day/once a day/once a week/less often)?

- Do you ever feel things that other people can’t? how often?

(many times an hour/ many times a day/once a day/once a week/less often)?

**Form/relationship:**

# Do you hear them in your head, or through your ears?

- Are there voices that you hear in your head, not from outside? How do you know they are not thoughts?
- What do the voices typically say? Example?
- Do you know the people? What is your relationship with the voices?
- Can you talk with them? Do they respond to you?

**Control:**

- Do you listen to what they say?
- How much do you pay attention?
- How much control do you feel that you have over them?
- Do they ever tell you what do to?
- Does it bother you when they tell you what to do?
- Do you do what they tell you?

**More about form and content:**

- Do you know the speakers?
- Who are the speakers: how old; what gender?
- Do they have any relationship to you? (friend/enemy/alien, etc.)
- Do the voices speak in only certain situations? When?
- Do the voices keep you awake at night?
- Are they present all the time, whether they are speaking or not?
- How do you know?
- Do they speak in the first or the third person?
- Is there only one voice at a time, or more than one voice, or many?
- Do the voices ever talk to each other? How often?
- Do they ever try to talk to people in the world through you?
- Do you talk to your parents about the voices? To your friends? Who knows that you hear voices? What do they say?

**Distressing:**

- Do you find them distressing?
- What do you find distressing about hearing the voices?
- Do they these voices refer to matters like masturbation or sex?
- Do you do anything to cope with them? [e.g.: deliberately ignore; distract yourself; speak; sing; read aloud]
- Do you ever talk to them and tell them to stop?
- Do you ever make deals with them?
- Do any of these methods work?
- Is it hard to pay attention to other things when the voice is speaking?
- Is there anything that triggers them?

**Positive things:**

- Are there voices that you like? Does the voice like you?
- Are the voices ever helpful?
- Are there voices that are connected with prayer?
- Does it ever cross your mind that the voices might be from God?
- Do you ever talk to your pastor/priest/guru about hearing voices?
- When you pray to God, does God ever talk back? What kind of things does God say?

**Realness:**

- What is the difference between you and the voices: are the voices part of you? If not how are they different? Do they know or want the same things as you?
- If your mother is in the room with you, do you think that she can hear the voices?
- Would you say that the voices are real or unreal? When you hear the voices, does it cross your mind that they are not real? Do they ever feel like make believe?

**Cause:**

- What causes these voices?

[biomedical /social tension / childhood trauma / spirits / masturbation/sex / other]

- What do you think caused your illness?

[biomedical /social tension / childhood trauma / spirits / masturbation/sex / other]

**Additional**-

1. Have you ever believed that you were being sent special messages through television or the radio, or that a program has been arranged just for you alone? Who are they coming from? Can you give an example?
2. Has anyone been making things hard, or purposely causing you trouble, or trying to hurt you, or plotting against you? How come? Could there be any other reason for it? What do your friends think about it?
3. Have you felt that you are a very important person or that you have special powers or abilities? What are they? Are you related to important people like kings or the prime minister or a sports figure? Can you do thing that others cannot do?
4. Have you ever felt that something was very wrong with you physically even though nothing was wrong… like you had cancer or some other terrible disease?
5. Have you ever been convinced that something strange was happening to parts of your body?
6. Have you ever felt that someone or something outside of yourself was controlling your actions against your will? Or that you are forced to say or do things that you don't want to?
7. Did you ever feel that thoughts that were not your own were put into your head? Who put them there? How? Why?
8. What about being taken out of your head? by someone or some special force? Tell me what happened. Why do you think this happened?
9. Did you ever feel as if your thoughts were being broadcast out so loud that other people could actually hear what you were thinking? Like on a radio, so that anyone listening could hear them?
10. When was the first time you ever experienced hallucinations? Probes – as a child perhaps? Once or twice? Or during your teen years?
11. Have you experienced any traumatic incidents? - did these hallucinatory experiences come before or after this incident?
12. When was the first time you heard the voices?
13. What do they talk to you about?
14. Have they always said these things, or have they said different things- explore details.
15. What would be the age of the voices be? Have they grown older or younger as you have?
16. What were some of your life experiences?
